# APOE‐ε4 is associated with poor sleep and structural differences of the hypothalamus in the PREVENT‐Dementia and ALFA cohort studies

**DOI:** 10.1002/alz.088305

**Published:** 2025-01-09

**Authors:** Axel AS Laurell, Elijah Mak, Benjamin R Underwood, Yves Dauvilliers, Craig W Ritchie, Ivan Koychev, Brian A Lawlor, Lorina Naci, Paresh Malhotra, Oriol Grau‐Rivera, Juan Domingo Gispert, John T O'Brien

**Affiliations:** ^1^ University of Cambridge, Cambridge, Cambridgeshire UK; ^2^ Université de Montpellier, Montpellier France; ^3^ Scottish Brain Sciences, Edinburgh UK; ^4^ University of Oxford, Oxford, Oxon UK; ^5^ Trinity College Dublin, Dublin Ireland; ^6^ Trinity College Institute of Neuroscience, School of Psychology, Trinity College Dublin, Dublin Ireland; ^7^ Imperial College London, London UK; ^8^ Barcelonaβeta Brain Research Center, Barcelona Spain; ^9^ Barcelonaβeta Brain Research Center (BBRC), Pasqual Maragall Foundation, Barcelona Spain

## Abstract

**Background:**

Sleep disturbances have been identified as a risk factor for developing dementia. The hypothalamus is involved in sleep regulation and may be affected early by neurodegeneration. Our aim was to investigate the relationship between subjective sleep and hypothalamic structure in adults at higher risk of developing dementia.

**Method:**

We analysed baseline data from PREVENT‐Dementia (n=700) and ALFA (n=1478), which are prospective cohort studies of cognitively healthy, middle‐aged participants, who were stratified by apolipoprotein E (APOE) genotype for their risk of dementia. Subjective sleep data was collected using the Pittsburgh Sleep Quality Index (PSQI), including the following components: sleep quality, latency, duration and efficiency. The volumes of five hypothalamic subunits were extracted by automatic segmentation of T1‐weighted 3T MRI images using Freesurfer (version 7.2.0). Combat harmonisation was used to account for using multiple MRI scanners. The data was analysed in R Studio using robust linear and logistic regression, correcting for age, sex, depression, total intracranial volume and multiple comparisons.

**Result:**

The ALFA cohort was significantly older than PREVENT‐Dementia (mean 60 versus 51 years, p<0.001). In the combined datasets, APOE‐ε4 homozygotes had a larger anterior‐superior hypothalamus compared to heterozygotes (p=0.02) and non‐carriers (p=0.02). Furthermore, APOE genotype moderated the association between age and volume of the anterior‐superior hypothalamus (p=0.02), such that APOE‐ε4 carriers had smaller volume with age (homozygotes: m=‐0.19, p=0.14; heterozygotes: m=‐0.17, p<0.01) compared with non‐carriers (m=‐0.10, p<0.01). Long sleep latency was associated with a smaller anterior‐inferior hypothalamus (p=0.05). Sleep latency also moderated the relationship between age and the volume of the anterior‐inferior hypothalamus (p=0.04). While subjects with short sleep latency showed a positive correlation between larger volume and age (m=0.06, p=0.07), there were no correlation with age in those with long sleep latency (m=‐0.01, p=0.91). In the ALFA cohort only, APOE‐ε4 carriers had longer sleep latency than non‐carriers (p=0.02).

**Conclusion:**

APOE‐ε4 carriers have a larger anterior‐superior hypothalamus and worse sleep, which may be due to early pathological processes. Our findings underscore the role of the hypothalamus in sleep homeostasis and suggest that involvement of subcortical structures may relate to early symptoms in individuals at risk of dementia.

